# Supplementary material for: Auditory cortex modulates call duration in rats
Source: Commun Biol. 2026 Jan 30;9:353. doi: 10.1038/s42003-026-09608-9 (PMC12976348; doi:10.1038/s42003-026-09608-9)
Supplement: Supplementary file 2 — Supplementary Information [file 42003_2026_9608_MOESM2_ESM.pdf]

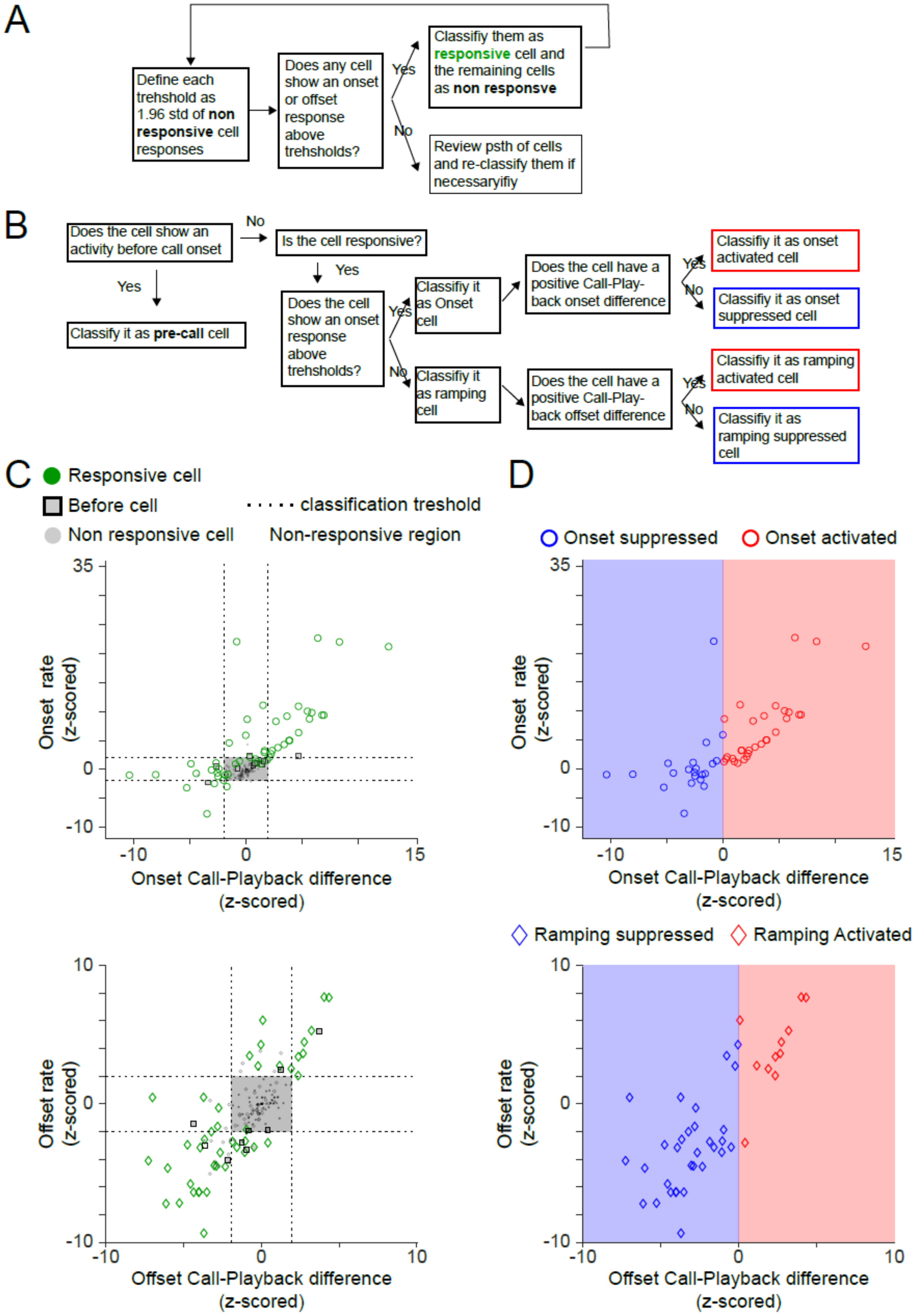

Supplementary Figure 1. Classification criteria for onset and ramping cells reveals distinct functional cell types.

A) Decision tree outlining the procedure for detecting responsive cells.

B) Decision tree illustrating the classification of responsive and non-responsive cells as pre-call, onset activated, onset suppressed, ramping activated or ramping suppressed cells.

C) Summary of the detection procedure results. Green labeled dots indicate cells labeled as responsive. Grey dots and squares indicated non-responsive cells at the end of the detection. Squares indicates cells further classified as pre-call cells. Shaded gray area indicates non-responsive region.

D) Summary of the classification results for responsive cells.

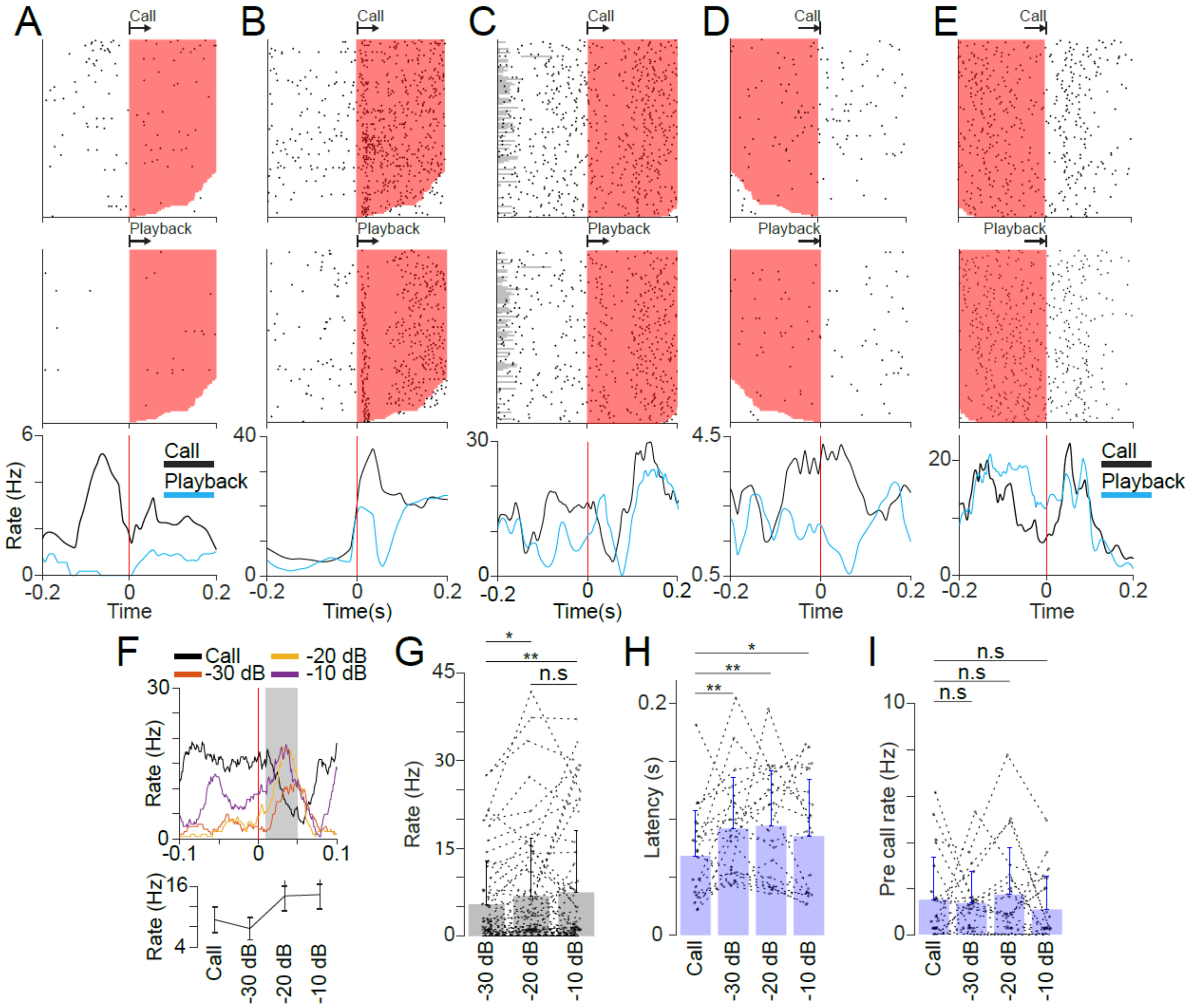

Supplementary Figure 2. Example cell types and profile of playback responses at different dB levels.

A) Raster plot and PSTH of an example pre-call neuron. Red shaded area represents the presence of calls or playback of calls.

B) Raster plot and PSTH of an example onset activated neuron.

C) Raster plot and PSTH of an example onset suppressed neuron.

D) Raster plot and PSTH of an example ramping activated neuron.

E) Raster plot and PSTH of an example ramping suppressed neuron.

F) Up: Example neuron showing different call and playback responses. Shaded gray area indicates time window to estimate call and playback responses as shown in panel G. Bottom: Mean call and playback responses of same cell, during the period indicated by the shaded region (between 10 ms and 50 ms after call onset).

G) Mean playback response rate of auditory cortex neurons for different playback dB levels.

H) Latency to call and playback of call responsive neurons (n=31) that showed a lower firing rate before call onset (rate lower than 10 Hz, n = 21). Latency was estimated as the mean elapsed time between the call or playback onset and the first spike.

I) Mean firing rate of responsive neurons with low pre-call firing rate (n=21) during the 100 ms window before call or playback onset. Error bars indicate the standard error of the mean (SEM).

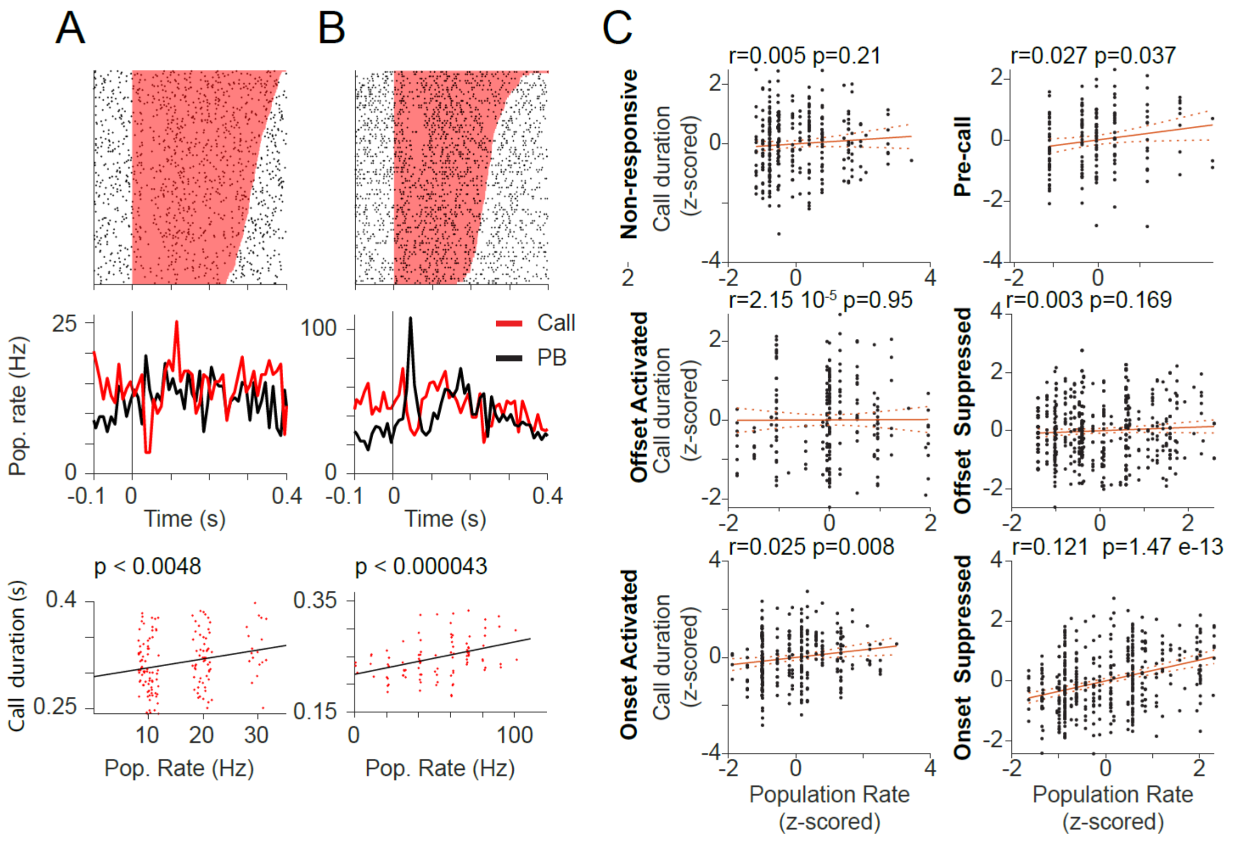

Supplementary Figure 3. Correlation between call duration and population rates for each auditory cortex cell type.

A) Same as on Figure 2 A-C, for a second animal. Upper. Raster plot of the activity of all onset suppressed neurons within one animal after call onset. Calls are sorted according to their duration. Mid: Population rate of all onset suppressed neurons within one experiment, in response to call (red) and playback (PB, black) onset. Bottom: The Population Rate during the 100 ms window before call onset is highly correlated with the duration of the upcoming call (black line represents linear regression fit. p-value of the corresponding Fisher test is indicated on top).

B) Same as on A, for a third animal.

C) Data of different animals was pooled together by z-scoring the population rate of a given cell type and the call durations in the current session. After pooling data from all animals, a linear regression between population rate and call duration was fit for each cell type.

Correlation value (r) and p value are reported on top of each panel. Dashed lines indicate confidence intervals.

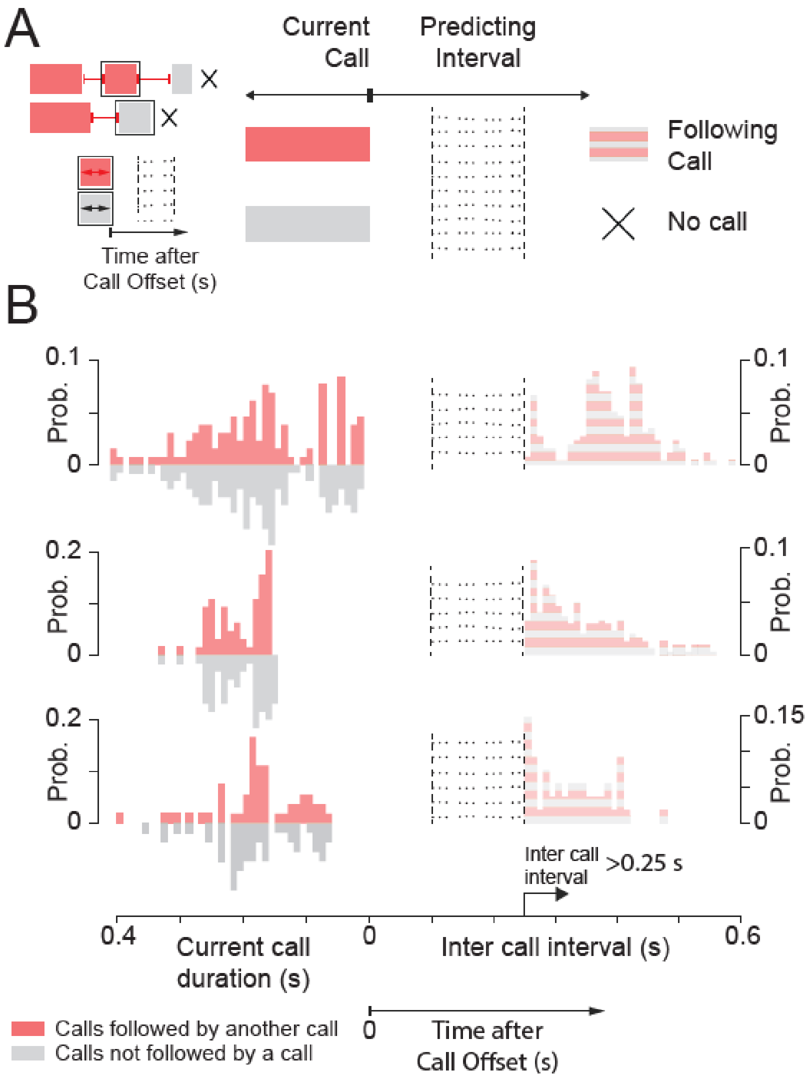

Supplementary Figure 4. Call selection procedure for predicting call occurrence.

A) Left: Diagram as on Figure 2A. Right: Prediction design. Current call duration is indicated as a left pointing arrow starting at call offset. The onset of the following call is indicated as a right pointing arrow starting at call offset. Predicting interval is located in a call free time period.

B) Up: Call selection procedure used to predict call occurrence as on Figure 2F. From the population of all calls followed by another call whose inter call interval was longer than 250 ms (n = 304), we selected a group matching the durations of the calls that were not followed by another call (n = 131). Figure shows the resulting paired distributions of call durations (red:

calls followed by another call; gray: calls not followed by another call. Kolmogorov test between the two distributions: $p = 0.91$). On the right (stripe pattern) the distribution of inter call intervals respect to the following call (following call onsets). Mid: Call selection procedure used to estimate rate and prediction for the second data set. From the population of all calls followed by another call whose inter call interval was longer than 250 ms ($n = 392$), a group matching the durations of the calls that were not followed by another call was selected ($n = 64$). Figure shows the resulting paired distributions (Kolmogorov test between the two distributions: $p = 0.38$). Bottom. Call selection procedure used to estimate rate and prediction for the third data set. From the population of all calls followed by another call whose inter call interval was longer than 250 ms ($n = 98$), a group matching the durations of the calls that were not followed by another call was selected ($n = 54$) Figure shows the resulting paired distributions (Kolmogorov test between the two distributions: $p = 0.05$).

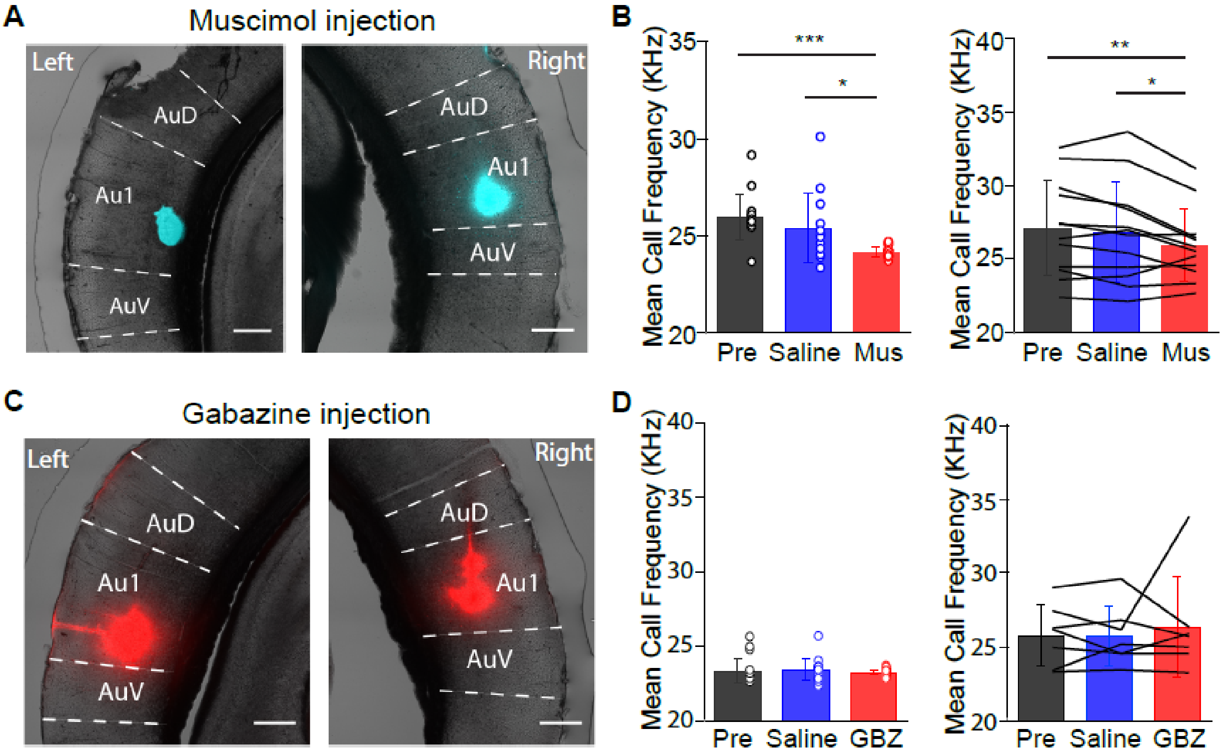

Supplementary Figure 5. The impact of injecting muscimol and gabazine into rat auditory cortex on the mean call frequency.

A) Post-hoc histology revealing the muscimol injection was primarily localized within Au1, the primary auditory cortex. AuD: secondary auditory cortex, dorsal area; AuV: secondary auditory cortex, ventral area. (Scale bars = 500μm)

B) Left: The mean call frequency of each trial from the example animal in Fig. 3B that were measured before (Pre) and after the injection of saline (Saline) and muscimol (Mus) into the auditory cortex, respectively. A one-way ANOVA showed a significant effect of the pharmacological manipulation [F (2, 40) = 8.851, P=0.0007]; a post-hoc Tukey's test showed that the mean call frequency in the muscimol treatment was significantly lower than that in the pre-treatment (p = 0.0005) and saline control treatments (p = 0.0247) (*p < 0.05, ***p < 0.001). Right: The summary of the mean call frequency of each trial from all the animals that were measured before (Pre) and after the injection of saline (Saline) and muscimol (Mus) into the

auditory cortex, respectively (n=12). A repeated measures one-way ANOVA showed a significant effect of the pharmacological manipulation [$F(2, 22) = 6.331$, $P=0.0067$]; a post-hoc Tukey's test showed that the mean call frequency in the muscimol group was significantly lower than that in the pre-treatment ($p = 0.0067$) and saline control treatments ($p = 0.0438$) ($^{*}p < 0.05$, $^{**}p < 0.01$).

C) Post-hoc histology revealing the gabazine injection was primarily localized within Au1, the primary auditory cortex. AuD: secondary auditory cortex, dorsal area; AuV: secondary auditory cortex, ventral area. (Scale bars = 500μm)

D) Left: The mean call frequency of each trial from the example animal in Fig. 3G that were measured before (Pre) and after the injection of saline (Saline) and gabazine (GBZ) into the auditory cortex, respectively. A one-way ANOVA showed no effect of the pharmacological manipulation [$F(2, 123) = 1.465$, $P=0.2351$]. Right: The summary of the mean call frequency of each trial from all the animals that were measured before (Pre) and after the injection of saline (Saline) and gabazine (GBZ) into the auditory cortex, respectively (n=7). A one-way ANOVA showed no effect of the pharmacological manipulation [$F(2, 12) = 0.2324$, $P=0.7961$]; Error bars indicate mean ± SD. ($^{*}p < 0.05$, $^{**}p < 0.01$, $^{***}p < 0.001$, $^{****}p < 0.0001$). Results are mean ± s.d.

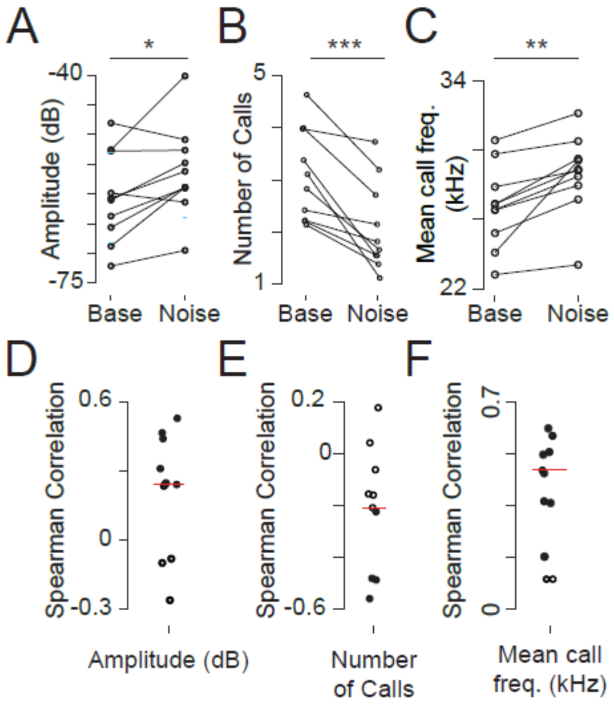

Supplementary Figure 6. Noise induces an increase in call amplitude and frequency and a reduction in call numbers.

A) Most rats show an increase in call amplitude during noise (relative dB values. See Methods). Each circle represents a different subject.

B) Rats consistently show a decrease in the number of calls during noise.

C) Rats consistently show an increase in call frequency during noise. (* = p<0.05, ** = p<0.01, *** p<0.001).

D) Most of the rats show a positive correlation between call amplitude and noise intensity level.

E) Most of the rats show a negative correlation between the number of calls and the noise intensity level, whereas only a minority are significantly correlated.

F) Most of the rats show a positive and significant correlation between call frequency and the noise intensity level (significant correlations are represented as filled circles).

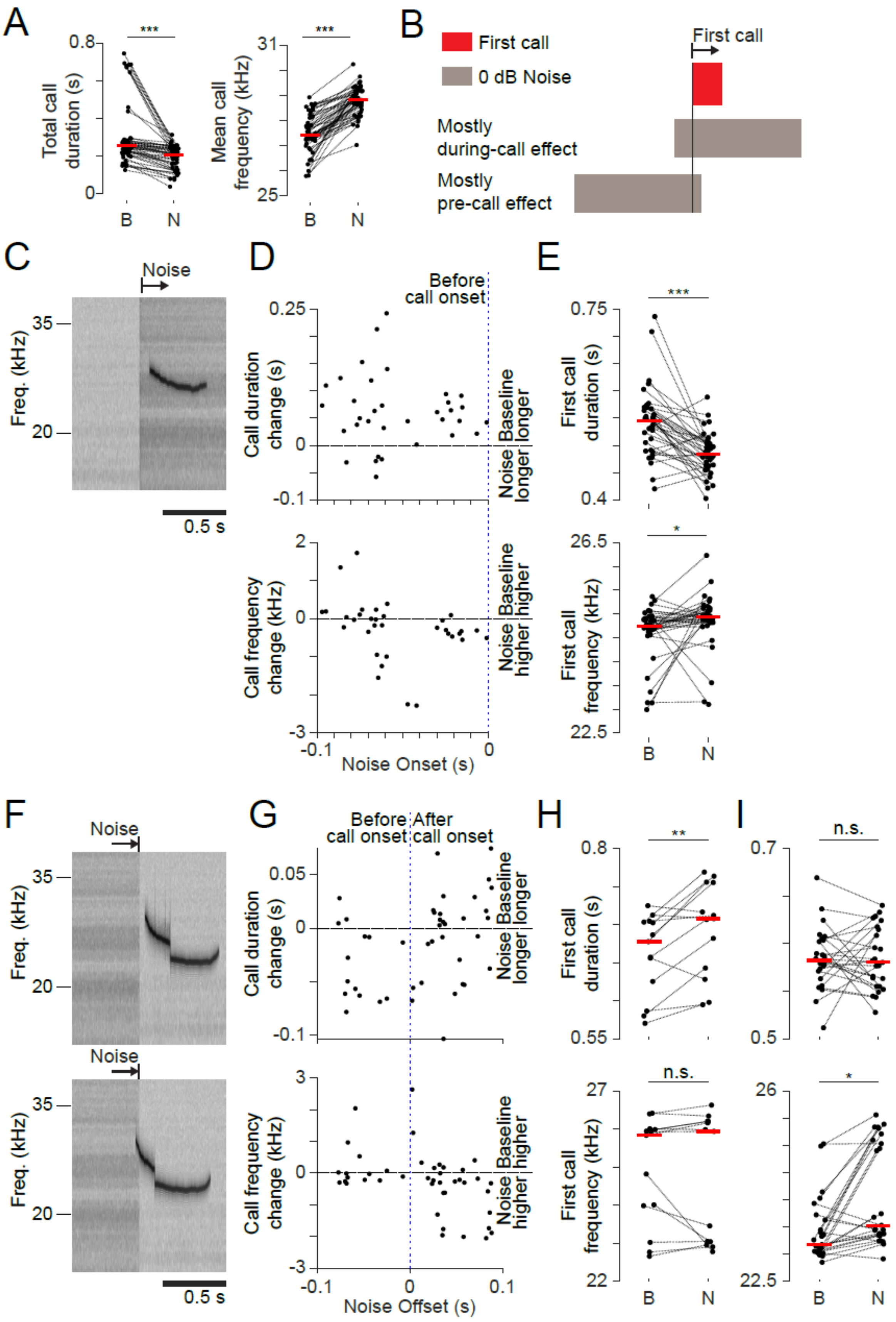
A
Total call duration (s)
0.8
0
***
B
N
Mean call frequency (kHz)
31
25
***
B
N
B
First call
0 dB Noise
First call
Mostly during-call effect
Mostly pre-call effect
C
Noise
Freq. (kHz)
35
20
0.5 s
D
Before call onset
Call duration change (s)
0.25
0
-0.1
Baseline longer
Noise longer
Call frequency change (kHz)
2
0
-3
Baseline higher
Noise higher
-0.1
0
Noise Onset (s)
E
First call duration (s)
0.75
0.4
***
First call frequency (kHz)
26.5
22.5
*
B
N
F
Noise
Freq. (kHz)
35
20
Noise
Freq. (kHz)
35
20
0.5 s
G
Before call onset
After call onset
Call duration change (s)
0.05
0
-0.1
Baseline longer
Noise longer
Call frequency change (kHz)
3
0
-3
Baseline higher
Noise higher
-0.1
0
0.1
Noise Offset (s)
H
First call duration (s)
0.8
0.55
**
First call frequency (kHz)
27
22
n.s.
B
N
I
0.7
0.5
n.s.
26
22.5
*
B
N

Supplementary Figure 7. Effect of timely delivered noise on call length, suggest different pre-call and during-call mechanisms.

A) Sustained noise stimulation consistently reduced first call duration (left) and increased first call frequency (right).

B) Experimental design. During-call condition: Noise stimulus was presented around the time of call onset and extended for 2 seconds. Pre-call condition: Noise stimulus was presented 5 seconds before call onset, and its duration was adjusted so that the call onset occurred around the time of noise offset. We analyzed changes in call duration and frequency induced by noise in the first call after PAG stimulation.

C) Example trial starting shortly before call onset. The first call after PAG stimulation occurred shortly after noise onset.

D) Top: Differences in duration between calls during noise and their corresponding baseline calls (baseline call duration minus noise call duration) are plotted against noise onset time relative to call onset. Positive values in the y axis indicate longer baseline calls. Bottom: Differences in frequency between calls during noise and their corresponding baseline calls (baseline frequency minus noise call frequency) are plotted against noise onset time relative to call onset. Positive values indicate higher frequency in baseline calls.

E) Top: Paired comparison of call durations during noise and baseline. Bottom: Paired comparison of call frequencies during noise and baseline.

F) Example trial eding shortly before (up) and after (bottom) call onset. The first call after PAG stimulation occurred around noise offset.

G) Top: Differences in duration between calls during noise and their corresponding baseline calls (baseline call duration minus noise call duration) are plotted against noise offset time relative to call onset. Positive values in the y axis indicate longer baseline calls. Bottom: Differences in frequency between calls during noise and their corresponding baseline calls

(baseline frequency minus noise call frequency) are plotted against noise onset time relative to call onset. Positive values in the y axis indicate higher frequency in baseline calls.

H) Top: Paired comparison of call durations during noise and baseline for negative values of noise offset (noise stimuli ending before call onset). Bottom: Paired comparison of call frequencies during noise and baseline for negative noise offsets.

I) Top: Paired comparison of call durations during noise and baseline for positive values of noise offset (noise stimuli ending after call onset). Bottom: Paired comparison of call frequencies during noise and baseline for positive values of noise offset. (* $p<0.05$, ** $p<0.01$, *** $p<0.001$)
