# Supplementary material for: Auditory cortex modulates call duration in rats
Source: Commun Biol. 2026 Jan 30;9:353. doi: 10.1038/s42003-026-09608-9 (PMC12976348; doi:10.1038/s42003-026-09608-9)
Supplement: Supplementary file 3 — Description of Additional Supplementary Files [file 42003_2026_9608_MOESM3_ESM.pdf]

# Description of Additional Supplementary Files:

**File name:** Supplementary Data 1

**Description:** The raw data of Figure 3 and Supplementary Figure 5.
